# A systematic review of EEG neurofeedback in fibromyalgia to treat psychological variables, chronic pain and general health

**DOI:** 10.1007/s00406-023-01612-y

**Published:** 2023-05-14

**Authors:** Carlos Barbosa Torres, Eloísa Julia Guerrero Barona, Mónica Guerrero Molina, María Elena García-Baamonde Sánchez, Juan Manuel Moreno Manso

**Affiliations:** University of Extremadura: Universidad de Extremadura, Badajoz, Spain

**Keywords:** Sensorimotor rhythms, Fibromyalgia, Anxiety, Depression, Pain

## Abstract

This paper is the first up-to-date review of the various EEG-neurofeedback treatments for fibromyalgia patients and their psychological, physiological and general health consequences. Searches were made of the PubMed, PsycNet, Google Scholar and Scopus databases according to PRISMA guidelines for empirical peer-reviewed articles on EEG-neurofeedback treatment of fibromyalgia, yielding a final selection of 17 studies that met the inclusion criteria: (1) published articles and doctoral theses; (2) conducted between 2000 and 2022; (3) reporting empirical and quantitative data. These articles show that there is a wide range of protocols with different designs and procedures to treat fibromyalgia using EEG-neurofeedback techniques. The main symptoms that showed improvement were anxiety, depression, pain, general health and symptom severity, whilst the most commonly used method was traditional EEG neurofeedback based on a sensorimotor rhythm protocol. It may be concluded from the review that the lack of consistency and uniqueness of the protocols makes it very difficult to generalise results, despite the individual improvements identified. This review provides instructions and information that could guide future research and clinical practise, with the data extracted helping to gain a deeper understanding of the state of the art and the needs of the technique for this population group.

## Introduction

Fibromyalgia is a chronic widespread pain syndrome associated with multiple symptoms ranging from chronic fatigue, sleep problems through to generalised hypersensitivity [1, 2]. In addition to physical problems, it should be added that the most frequent psychological factors in fibromyalgia patients include intense negative emotions (anxiety, depression), inadequate coping strategies, an excessive worry response and a maladjusted attention pattern [3] resulting in low or no quality of life [4].

This syndrome affects up to 5.0% of the world population, mainly in women [5, 6]. A lack of knowledge of the exact cause, the absence of biomarkers and the great variety of signs and symptoms make its diagnosis very difficult, despite numerous studies worldwide [7]. The most widely held hypothesis is currently based around Central Sensitisation Syndromes (CSS), which involves increased sensitivity of the central nervous system and decreased perceptual thresholds [8].

The current treatment of fibromyalgia is symptomatic and multidisciplinary, combining pharmacological and non-pharmacological treatments [9]. The main treatment chosen by patients continues to be pharmacological, such as amitriptyline, or other tricyclic antidepressants, even though they are not explicitly prescribed for people with fibromyalgia [10]. Although the recommended treatment includes the use of medication of various kinds, along with moderate physical exercise and psychological therapy [11, 12], technological advances in neuroimaging since the 1970s have brought about the use of different neurofeedback techniques as symptomatic therapy in the treatment, amongst other symptoms, of pain, insomnia and depression [13]. However, even now, any kind of neurofeedback as treatment is still not used as a generalist therapy for fibromyalgia [14, 15].

One of the most commonly used techniques of neurofeedback is EEG neurofeedback, which is a non-invasive feedback treatment that seeks to obtain information on patients’ brain activity in real time, allowing direct modifications to that activity to improve certain symptoms without any side effects [16]. EEG neurofeedback involves the use of a specific neuroimaging technique called electroencephalography (EEG). It is one of the most accessible and affordable techniques, which consists of placing one or multiple electrodes on the patient’s scalp to measure various parameters of cortical activity such as amplitude, frequency and/or coherence of the EEG signal [17]. The raw signal is the result of the activity of millions of neurons in the cerebral cortex located below the electrode. The EEG-neurofeedback signal is analysed using software which provides feedback to the patient via an auditory or visual signal so that they can learn to control and shape brain patterns [18]. Traditional neurofeedback is based on operant conditioning [19]; whilst in alternative neurofeedback, the patient does not receive any direct feedback signal, with the EEG receiving the feedback to compensate the patient’s dominant frequency [20]. Both forms of neurofeedback have been used for multiple purposes aimed at changing the brain activity underlying psychological [21], physiological and general health problems associated with the disease [22]. Some researchers have questioned whether neurofeedback has any beneficial effect on pain or other problems, attributing their findings to placebo effect or outcome expectancy [23, 24]. Nevertheless, there are many studies that have used one of the versions of neurofeedback for several types of chronic pain, in addition to patients with fibromyalgia, such as those suffering from migraine, central neuropathic pain, chronic spinal cord injury, multiple sclerosis or post-herpetic neuralgia [25].

In light of the above, the main objectives of this review are to review the current status of the efficacy of different types of EEG neurofeedback on fibromyalgia and to evaluate the effect of EEG-neurofeedback interventions for fibromyalgia patients to treat symptoms associated with the disease, such as physiological and general health problems (chronic pain and fatigue), psychological problems (anxiety and depression), neuropsychological problems (attention) and their effect on brain activity.

## Methods

A systematic literature review was conducted following the Preferred Reporting Items for Systematic Reviews and Meta-Analyses (PRISMA) guidelines [26].

### Inclusion and exclusion criteria

All articles with samples composed of patients with fibromyalgia were considered eligible for this review. The following criteria were used to determine whether studies were eligible for inclusion: (1) scientific articles or doctoral theses; (2) carried out until 2022; and (3) reporting quantitative data. Only empirical research studies were considered (not theoretical reviews, nor books nor book chapters) involving use of EEG neurofeedback for patients with fibromyalgia. The main measures of interest were psychological and neuropsychological disorders, symptom severity, sleep quality, pain and general health. There were no sociodemographic restrictions.

Articles in which the title, abstract or keywords did not refer to the study subject were excluded from the study. Studies in which the main biofeedback training procedure was not EEG neurofeedback, such as heart rate variability or electromyographic biofeedback, were also not considered.

### Data sources and search strategy

The search was carried out in the PubMed, Google Scholar, PsycNet and SCOPUS databases in February 2023.

The eligibility criteria were kept broad to encompass the range of empirical studies of EEG-neurofeedback treatment in the sample. The algorithm used for papers in each database was as follows: e.g. Search "neurofeedback in fibromyalgia" [OR] "neurofeedback training in fibromyalgia" [OR] "neurofeedback protocol in fibromyalgia"[AND] "neurotherapy" [AND] "electroencephalography" [OR] "EEG". The references of eligible studies and relevant reviews were also searched using a snowballing technique. The reference data were retrieved and duplicates were subsequently eliminated.

**Fig. 1:**
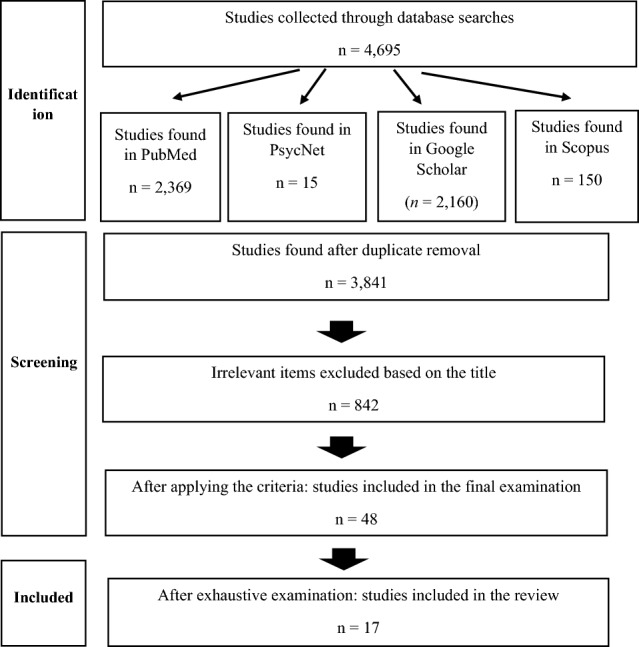
PRISMA flowchart

During the selection process, two independent reviewers (E.G. and J.M.M.) performed quality assessment and all duplicate articles were identified and eliminated. The risk of bias in the eligible studies was evaluated using the Newcastle–Ottawa Scale for cross-sectional studies [27], which consists of a rating system that can be used to compare studies according to various dimensions of the design, analysis and presentation of findings. Finally, the articles that met the inclusion criteria after screening the title and abstract were retrieved and read in full to reach a final decision. The entire process of identification, searches and inclusion can be seen in Fig. 1.

#### Data extraction and analysis

For each study, the following data were extracted: article title, name of the authors and year of publication, region/country where the study was conducted, study design, gender and average age of the participants, sample selection, diagnostic criteria, type of EEG neurofeedback, measurements collected, complementary therapies, group comparison measure, timing and number of sessions, and if they performed follow-up of the patients. Any disagreements were resolved following discussion amongst the reviewers and team consensus.

## Results

### Description of selected studies

Following an exhaustive examination, a final total of 17 articles reviewed and included was obtained. Table 1 shows the authors, patient characteristics and study design of all the articles.

**Table 1:** Data on authors, patient characteristics and study design

Authors (year)	Country	Sample size	Sex [%]	Age M [SD]	Patient recruitment	Study design
Donaldson et al. [28]	Canada	A: 157	Female [78.3]	A: 44.2	Multidisciplinary clinic referral	Retrospective study
Mueller et al. [29]	Canada	A: 30	Female [90]	50.7 [12.0]	Multidisciplinary clinic referral	Pre-post cohort study
Kravitz et al. [30]	USA	A: 33 B: 31	Female [92]	46.9 [9.0]	Private practice referral; community advertisement	Randomised double-blind placebo-controlled study
Kayiran et al. [31]	Turkey	A: 3	Female [93]	A: 32 [1.0]	Derived from Medicine and Rehabilitation Outpatient Clinic	Case study
Kayiran et al. [32]	Turkey	A: 20 B: 20	Female [100]	A: 31.8 [6.2] B: 32.4 [6.7]	Derived from Medicine and Rehabilitation Outpatient Clinic referral	Randomised, rater-blind controlled study
Nelson et al. [33]	USA	A: 17 B: 17	Female [97]	A: 51.6 [8.6] B: 52 [11.4]	Fibromyalgia database of OHSU Division of Arthritis and Rheumatic Diseases; community advertisement	Randomised double-blind placebo-controlled study
Caro & Winter, [34]	USA	A: 15 B: 63	Female [100]	A: 66.7 [12.3] B: 50.5 [13.9]	Derived from Outpatient Rheumatology Office	Pre-post cohort study with comparison with historical control group
Kristevski et al. [35]	USA	A: 5	Female [100]	A: 36.2 [12.96]	Recruited through the pain management services of several Northshore Integrative Healthcare (NIH) units	A case-series design in which each participant’s data were analysed separately
Barbosa et al. [36]	Spain	A: 40	NR	NR	NR	Pre-post cohort study
Goldway et al. [37]	Israel	A: 25 B: 9	A: Female [96] B: Female [77.78]	A: 35.5 [12.6] B: 35.9 [10.6]	Institute of Pain Medicine at Tel Aviv Medical Center in Israel	Repeated measures with comparison with sham control group
Barbosa et al. [38]	Spain	A: 40	Female [90]	A: 31–70	Derived from Pain Unit of the Spanish National Health Service	Pre-post cohort study
Farnia et al. [39]	Iran	A: 22 B: 22	Female [100]	A: 41.64 [5.70] B: 39.75 [5.06]	Recruited through Tuba Specialized Clinic and Imam Khomeini Hospital in Sari	Pre-post cohort study
Pérez-Elvira et al. [40]	Spain	A: 1	A: Female [100]	A: 37	NEPSA Neurological Rehabilitation, a clinic licenced by the National Health Service	Single case study
Terrasa et al. [41]	Spain	A: 17	Female [100]	A: 54.94 [10.11]	Derived from the Fibromyalgia Syndrome Association	Pre-post cohort study with comparison and control group
Barbosa & Cubo, [42]	Spain	A: 37	Female [100]	A: 54.92 [7.89]	Derived from the Pain Unit of the Spanish National Health Service	Quasi-experimental study of a single group with pre- and post-tests
Barbosa & Cubo, [43]	Spain	A: 37	Female [100]	A: 54.92 [7.89]	Derived from the Pain Unit of the Spanish National Health Service	Quasi-experimental study of a single group with pre- and post-tests
Wu et al. [44]	Taiwan	A: 60 B: 20	Female [95]	A: 48.6 [13.5] B: 42.2 [10.9]	Recruited by referral from physicians	Randomised controlled trial with comparison and control group

*NR* not reported, *SD* standard deviation, *CG* Control group

### Description of the types of EEG-neurofeedback protocols

Most of the studies used traditional neurofeedback rather than alternative neurofeedback (12 out of 17 studies). Of the eight different types of EEG-neurofeedback protocols identified and described, three correspond to traditional neurofeedback [Sensorimotor Rhythm Protocol (SMR), alpha rhythm feedback, alpha-theta neurofeedback] whilst five consisted of alternative EEG neurofeedback [Flexyx neurotherapy system, EEG-Driven Stimulation (EDS), Low-Energy Neurofeedback (LENS), Amyg-EFP-neurofeedback and standardised low-resolution brain electromagnetic tomography (sLORETA)]. Table 2 shows the type of EEG neurofeedback and its effectiveness, the instruments and the characteristics of the session in chronological order.

**Table 2:** Protocol and session data

Authors (year)	Type neurofeedback	Channels	Psychological, neuropsychological and medical measures	Psychological, neuropsychological and medical outcomes	EEG outcomes	Monotherapy [Y/N]	Comparison group	Length of treatment sessions	Number of sessions	Follow-up
Donaldson et al. [28]	Flexyx neurotherapy system—alternative	Electrode cap	+ Visual Analogue Scales (VAS) for sleep disturbance and pain + Symptom Checklist of fibro-fog and emotional factors + Fibromyalgia Impact Questionnaire (FIQ)	Improvements in cognitive functioning and quality of sleep, although the intensity of pain increased in the parts of the body that were reported as painful after the intervention	No specific changes indicated	N [massage therapy, physical therapy, neuromuscular retraining]	None	NR	4–6 months	None
Mueller et al. [29]	EEG-Driven Stimulation (EDS)—alternative Delta, theta, alpha, beta1 and beta2	21 scalp sites [FP, FP1, F7, T3, T5, O1, O2, T6, T4, F8, FP2, F3, C3, C3, P3, P4, C4, F4, Fz, Cz, Pz and Oz]	+ Fibromyalgia Impact Questionnaire (FIQ) – Symptom Checklist 90 items—Revised + Visual Analogue Scales (VAS) for pain intensity, sleep, fatigue, cognitive clouding, depression and anxiety	Notable improvements were observed in terms of pain intensity, cognitive processing difficulties, mood, sleep and (to a different but less marked degree) tiredness and fatigue	Delta, high, theta and low alpha were found with greater frequency in FP, FP1, FP2, Fz, F3, F4, F7, F8, Cz, C3 and C4 (The biggest difference between pre- and post-treatment was in delta, generally more anterior and central)	N [pharmacological treatment; massage; physiotherapy; electromyography]	None	1 h	Varied *M* = 51 h SD = 23.6 [3–5/week to 1–2/week]	8.2 months [4.3]
Kravitz et al. [30]	LENS [Low-Energy Neurofeedback System]—alternative Delta/alpha protocol	21 scalp sites	+ Clinical Global Impressions Scale (CGI-I) and participant- (PGI-I) + Seven Likert-type scales measuring pain (generalised and specific), memory, concentration, multitasking, depression and fatigue + Symptom Checklist-90-R (SCl-90-R) + Fibromyalgia Impact Questionnaire (FIQ) + Dysfunction Questionnaire (CNS)	No significant differences were found between groups in the SCl-90-R, nor in the FIQ	There were no significant differences between the neurofeedback and placebo groups in terms of EEG amplitude change (means and standard deviations) from pre-treatment to the final treatment session	Y	Sham FNS	NR	22 [2/week]	1 week
Kayiran et al. [31]	Sensorimotor rhythm Protocol—traditional [SMR, theta and theta/SMR]	Monopolar [C4]	+ Visual Analogue Scale (VAS) for pain + VAS for fatigue + Short Form 36 (SF-36) + Hamilton Depression Scale (HDS) + Beck Depression Scale (BDS) + Hamilton Anxiety Scale (HAS) + Beck Anxiety Scale (BAS)	Significant reduction in fibromyalgia symptoms, reduction in pain intensity, less difficulty in cognitive processing, improved mood, sleep and fatigue	A significant decrease in the theta/SMR ratio. Increase in SMR values and reduction of theta values	Y	None	30 min	10 [3/week]	None
Kayiran et al. [32]	Sensorimotor rhythm Protocol—Traditional [SMR, theta and theta/SM]	Monopolar [C4]	+ Visual Analogue Scale (VAS) for pain + VAS for fatigue + Short Form 36 (SF-36) + Hamilton Depression Scale (HDS) + Beck Depression Scale (BDS) + Hamilton Anxiety Scale (HAS) + Beck Anxiety Scale (BAS) + Fibromyalgia Impact Questionnaire (FIQ)	Significant reduction in pain and fatigue, significant improvements in depression, anxiety and fibromyalgia symptoms and improvement in the impact of the disease	A significant decrease in the theta/SMR ratio. Increase in SMR values and reduction of theta values	Y	Medication only group [10 mg/day of escitalopram treatment for 8 weeks]	30 min	20 [5/week]	2 weeks 4 weeks 8 weeks 16 weeks 24 weeks
Nelson et al. [33]	LENS [Low-Energy Neurofeedback System]—alternative	19 scalp electrode sites	+ Fibromyalgia Impact Questionnaire (FIQ) + Quantitative Sensory Testing (QST) + Profile of Mood States Bi-Polar Form Clearhead Confused Scale (POMS BI-CC) + Brief Fatigue Inventory (BFI) + Medical Outcomes Study Sleep Scale (MOS-Sleep) + Patient Health Questionnaire-9 (PHQ-9)	There was no significant result for the impact of fibromyalgia, but there were changes for both groups in terms of self-reported cognitive dysfunction, fatigue and global distress	No specific changes indicated	Y	Sham therapy group	NR	22 [2/week]	3 months 6 months
Caro & Winter, [34]	Sensorimotor Rhythm Protocol—traditional [SMR, theta and high beta]	Monopolar [Cz]	+ Test of Variables of Attention (TOVA) + Laboratory testing (serial measurements of pain, fatigue, psychological distress, morning stiffness and tenderness) + Visual and auditory continuous performance testing (CPT)	Improvement in attentional processes, global pain and fatigue reduction	No specific changes indicated	Y	Historical control group	NR	Varied *M* = 58 SD = 40–98	None
Kristevski et al. [35]	Sensorimotor Rhythm Protocol—traditional (SMR, theta and high beta)	Monopolar [C4]	+ Revised Fibromyalgia Impact Questionnaire (FIQR) + Visual Analogue Scales (VAS) for pain + ME/CFS Fatigue Types Questionnaire (MFTQ)	Improvement in symptoms associated with fibromyalgia and reduction of symptoms of fatigue and pain	Decreased theta and increased SMRs in the majority of patients	N [the simultaneous treatments were not specified]	Wait-list control	30 min	Between 8 and 16 sessions [4–8 weeks]	None
Barbosa et al. [36]	Sensorimotor Rhythm Protocol [SMR/theta]—traditional	Monopolar [C4] [C4	+ Trail Making Test (TMT) + Visual Analogue Scale (VAS) for pain + SF-36 Health Survey	Improved reaction times in attention tests, improved perception of general health and subjective perception of pain	No specific changes indicated	Y	None	NR	20	None
Goldway et al. [37]	Amyg-EFP- Neurofeedback—alternative	Electrode cap	+ Visual Analogue Scale (VAS) for pain + Fibromyalgia Impact Questionnaire (FIQ) + Beck Depression Inventory (BDI) + Trait Anxiety Inventory (STAI-T) + Pittsburgh Sleep Quality Index (PSQI)	Improved perception of sleep, anxiety and depression which predicted the effects of long-term pain improvement	No specific changes indicated [Amyg-EFP-neurofeedback aimed at reducing limbic modulation]	N [pharmacological treatment]	Sham group	NR	10 [5 weeks]	16.2 months [8.72]
Barbosa et al. [38]	Sensorimotor Rhythm Protocol [SMR/theta]—traditional	Monopolar [C4] [C4	+ Beck Depression Inventory (BDI) + State-Trait Anxiety Inventory (STAI)	Improvement in depression and anxiety scores after therapy	No specific changes indicated	Y	None	NR	20 [7 weeks]	None
Farnia et al. [39]	Alpha-theta neurofeedback and sensorimotor rhythm neurofeedback—Traditional	Monopolar—[Cz for SMR neurofeedback & Pz for alpha–theta neurofeedback]	+ Visual Analogue Scale (VAS) for pain + Multiple Outcomes Study (MOS) Sleep Scale + Fibromyalgia Impact Questionnaire (FIQ)	Improved pain perception and improved sleep	No specific changes indicated [The use of alpha-theta neurofeedback is similar to SMR neurofeedback, and both could be a suitable method to reduce FM symptoms]	N [Pharmacological treatment; 50–150 mg of pregabalin]	None	30 min	8 [4 weeks]	> 8 weeks
Pérez-Elvira et al. [40]	sLORETA Neurofeedback [theta]—alternative	19-channels [Fp1, Fp2, F7, F3, Fz, F4, F8, T3, C3, Cz, C4, T4, T5, P3, Pz, P4, T6, O1 and O2]	+ Beck Anxiety Inventory (BAI) + Beck Depression Inventory-II (BDI-II) + Symptom Checklist 90-R (SCL-90-R) + Analogue Visual Scale (VAS) for pain	Significant results were observed for pain, depression, anxiety and associated psychopathology following treatment	Reduction in the amplitude of theta	Y	None	30 min	5 [15 days]	None
Terrasa et al. [41]	Sensorimotor Rhythm Protocol—traditional [SMR]	21 scalp sites	+ Beck Depression Inventory (BDI-II) + Short Form 36 (SF-36) + West Haven-Yale Multidimensional Pain Inventory (WHYMPI) + McGill Pain Questionnaire (MPQ) + Pain Anxiety Symptoms Scale (PASS) + Tampa Scale for Kinesiophobia (TSK) + Pain Vigilance and Awareness Questionnaire (PVAQ) + Coping Strategies Questionnaire (CSQ) + MOS Social Support Survey (MOS) + Fibromyalgia Impact Questionnaire (FIQ)	Good SMR responders improved perceived pain and quality of symptoms associated with fibromyalgia	Increased SMRs in good SMR responders There was no modulation of SMR power in either the bad responders or the placebo group	Pharmacological treatment (analgesic/myorelaxant (88.24%), antidepressant (76.47%) and anxiolytic (70.59%)	Good SMR responders, bad SMR responders and control group that received false feedback during the training task (sham)	Maximum time of 40 min	6 [3/week]	None
Barbosa & Cubo, [42]	Sensorimotor Rhythm Protocol, [SMR/theta]—traditional	Monopolar [C4]	+ Chronic Pain Scale (CPS) + Short Form 36 (SF-36) + Pittsburgh Sleep Quality Index (PSQI)	Improvements in perceived pain, general state of health and sleep quality	Increase in SMR amplitude and decreased theta amplitude; significant increase of SMR/theta in the somatosensory cortex	Y	None	15 min	20 [3 weeks]	None
Barbosa & Cubo, [43]	Sensorimotor Rhythm Protocol—traditional [SMR/theta] [SMR/theta	Monopolar [C4]	+ Revised Fibromyalgia Impact Questionnaire (FIQR) + Visual Analogue Scale (VAS) + General Health Questionnaire (GHQ-28)	Significant results were observed for symptoms associated with fibromyalgia, with improved perceived pain and general state of health	Increase in SMR amplitude and decrease in theta band amplitude; significant increase in SMR/theta throughout the training; increase in SMR/theta ratio in three of the four pre-test/post-test conditions	Y	None	15 min	20 [3 weeks]	None
Wu et al. [44]	Alpha rhythm feedback [2 weeks]; Sensorimotor Rhythm Protocol—traditional [4 weeks]; choice between the two [2 weeks]	Bipolar [C3, C4and Cz]	+ Brief Pain Inventory (BPI) + Fibromyalgia Impact Questionnaire-Revised (FIQR) + Pittsburgh Sleep Quality Index (PSQI) + Psychomotor Vigilance Test (PVT) + Digit Span Tests (DSTs)	Significant improvements in sleep latency, pain and sustained attention compared to patients in the control group	They do not indicate specific changes of any of the protocols during the intervention	Y	Telephone support group—health educational treatment	30 min	20 [8 weeks]	None

## Discussion

This article reviews the available evidence regarding the efficacy of EEG neurofeedback as a treatment for fibromyalgia and its impact on psychological, physiological and general health variables. This is the first up-to-date systematic review of the use and effects of EEG neurofeedback as a treatment for fibromyalgia and associated symptoms.

### The EEG-neurofeedback protocols studied

The impact on the EEG of patients at the neurophysiological level varied greatly depending on the protocol and the measurement channels used, although we observed that in a high percentage of the studies (47.05%), the changes in brain activity were not assessed (Barbosa et al. [36, 38]; Caro and Farnia et al. [39]; Donaldson et al. [28]; Goldway et al. [37]; Nelson et al. [33]; Winter, [34]; Wu et al. [44]). Of the studies that do assess brain activity, not all of them analyse the same variables under the same conditions, meaning that the results depend on the protocol used.

Many of these protocols had common elements such as the location of the measurements, with the somatosensory area (C4; C3) being one of the most commonly used areas, especially in traditional EEG neurofeedback. Also, six of the studies used helmets with a varying number of electrodes (19–21). However, most of the studies did not use exactly the same protocol, except for Barbosa et al. [36, 38], Barbosa and Cubo [42, 43], Donaldson et al. [28] and Kayiran et al. [31, 32]. The most common measurement and feedback frequencies were SMR and theta due to the high number of studies using an SMR protocol (Kayiran et al. [31, 32]; Caro and Winter [34]; Kristevski et al. [35]; Terrasa et al. [41]; Barbosa and Cubo [42, 43]). The large number of studies with this SMR protocol may be due to its scientific relevance dating back to the 1960s [45]. This is not the case of the other protocols, with newer forms of EEG neurofeedback remaining largely unused in research on fibromyalgia and other forms of chronic pain until well into the twenty-first century [46]. In all the studies, SMRs increased and theta waves significantly decreased their frequency. An exception is the bad SMR responders in Terrasa et al. [41], for whom there was no SMR power modulation. Pérez-Elvira et al. [40] used a theta protocol, which also reduced the amplitude of this wavelength. The third most common feedback wavelength was delta. The studies by Kravitz et al. [30] with a delta/alpha protocol found no significant differences between the experimental group and the placebo group, whilst Muller et al. [29] studied various wavelengths and found an increase in delta frequency, especially in the central and anterior regions.

Following the above, it can be determined that the EEG-neurofeedback studies have not presented unanimity in their protocols.

### The design of EEG-neurofeedback studies

The protocols have been complemented by different designs that vary greatly depending on the number and length of sessions. The number of sessions ranges from 5 (Pérez-Elvira et al. [40]) up to 51 sessions on average (Muller et al. [29]) and in terms of the duration they are divided into 1 h sessions (Muller et al. [29]), 30–40 min sessions (Farnia et al. [39]; Kayiran et al. [31, 32]; Kristevski et al. [35]; Pérez-Elvira et al. [40]; Terrasa et al. [41]; Wu et al. [44]) or 15 min sessions (Barbosa & Cubo, [42, 43]. The rest of the studies do not provide data in this respect (Barbosa et al. [36, 38]; Caro and Winter [34]; Donaldson et al. [28]; Goldway et al. [37]; Kravitz et al. [30]; Nelson et al. [33]). Some studies assessed whether there were changes in brain activity over the course of the training sessions (Kayiran et al. [32]; Kristevski et al. [35]), whilst some assessed changes in brain activity before and after the treatment in a state of rest (Kayiran et al. [31]; Mueller et al. [29]; Pérez-Elvira et al. [40]; Terrasa et al. [41]) and others assessed them before starting treatment and during the last session (Kravitz et al. [30]). In addition, without excluding any of the above conditions, different conditions have also been assessed depending on the cognitive activity of the patient at the time of measurement (Barbosa and Cubo [42, 43]; Goldway et al. [37]).

In relation to the treatments, the studies that did not use EEG neurofeedback as a monotherapy were by Muller et al. [29] and Donaldson et al. [28], who did not specify interaction with EEG neurofeedback. However, Muller et al. [29] explained that once they had considered that the targets for change had been met with EDS therapy, they reduced the sessions to one or two per week and added body massage therapy, physiotherapy and surface electromyography (sEMG) to the treatment. These treatments were added to address localised pain, as well as the patients’ balance, posture and myofascial tension. The authors noted that the cognitive improvements induced by EDS allowed the patients to participate in these treatments for the first time. Golway et al. [37], Farnia et al. [39] and Terrasa et al. [41] included other pharmacological treatments in their studies at the same time as EEG-neurofeedback treatment. Finally, Kristevski et al. [35] did not specify the type of simultaneous treatment in their study. In light of the above, future studies should consider designs to test whether or not psychological, physiological and general health improvements are enhanced by EEG neurofeedback as a stand-alone therapy or as a complementary therapy. One of the few aspects on which all the studies agree is the type of reinforcement, given that due to the nature of the techniques, the options for presenting the reinforcing stimuli were either visual or auditory in all cases. After this review, the great variability of designs in EEG-neurofeedback studies can be highlighted, making it difficult to generalise the results.

### The mechanisms underlying EEG-neurofeedback treatment

The main goal of neurofeedback is to change brain activity that is believed to underlie or influence certain symptoms; however, each form of EEG neurofeedback uses different tools to achieve this goal. All traditional neurofeedback training has certain common elements. The SMR protocol consists of changing the firing patterns of sensorimotor rhythms from rapid and non-rhythmic to rhythmic and systematic [47] SMR training also increases the amplitude and elongates the latency of P300, thus facilitating thalamocortical inhibitory mechanisms [48]. The alpha–theta neurofeedback protocol aims to increase the subject’s ratio of theta to alpha waves to reach a state of physiological relaxation similar to sleep states [49, 50], whilst the alpha rhythm feedback protocol consists of the reduction of alpha waves to improve cognitive performance [51]. These three types of EEG neurofeedback work with operant conditioning, whereas alternative neurofeedback treatments work within a classical conditioning paradigm [52]. The alternative treatments differ from traditional forms of EEG neurofeedback in that they do not involve active participation by the patient to alter the feedback stimulus, i.e. no attention or learning activities are imposed on the patient, meaning that the process of change begins immediately and changes seem to occur more quickly. Of those considered in the review, the EEG-Driven Stimulation (EDS) monitors the patient’s EEG in the 0–30 Hz frequency band and uses the dominant frequency to set the frequency of light stimulation that is fed back to the patient. The difference between the dominant and stimulation frequency can be increased or decreased as needed in the therapy [53]. Low-Energy Neurofeedback (LENS) is a method designed to obtain constant measurements of cortical activity and allows small changes to the electromagnetic frequency at 19 or more locations. This input stimulation varies from one moment to the next and is updated according to the dominant frequency changes of the EEG [54]. Another variant of the EEG-neurofeedback therapy carried out by Donaldson et al. (1998), which also involves brain stimulation by means of an external energy source, such as electromagnetic energy (EM) through the connection cables of the connection of the connection of the EEG, is known as the Flexyx (FNS) neurotherapy system. Another method used in the review was Amyg-EFP-neurofeedback, which is based on an fMRI-driven EEG computational model that reflects the activation of the limbic system (i.e. the amygdala and supporting regulatory networks) via a simultaneous EEG/fMRI recording known as the Amygdala-electrical fingerprint (Amyg-EFP) to improve regulation of the amygdala-BOLD (blood-oxygen-level dependent) signal and functional connectivity [55]. Finally, the standardised low-resolution brain electromagnetic tomography (sLORETA) can be used to calculate statistical maps of EEG and magnetoencephalography (MEG) data that indicate the locations of underlying source processes with very low error. In the case of the only study that used this method [40], it was aimed at inhibiting the amplitude of the theta frequency band.

### Efficacy of EEG neurofeedback in fibromyalgia patients

In all the studies, the principal participants were women (77.78–100%) with an average age of 42.9 years. This fits the populational characteristics that are typically described for the fibromyalgia syndrome [56]. In addition, the overwhelming majority of fibromyalgia patients seeking medical care are women [57]. Likewise, our review shows significant improvements in those main variable measures that define the disease [58]. The main outcomes were improvement in pain, general health and symptom severity, along with anxious and depressive symptoms, except in the case of the studies by Kravitz et al. [30] and Nelson et al. [29], which found no significant results in terms of the impact on fibromyalgia, and Pérez-Elvira et al. [40] which found no significant results for pain, depression and anxiety. Other less frequently reported secondary measures such as psychopathological symptoms, attention and sleep also showed significant improvement, with the exception of the studies by Nelson et al. [29] and Pérez-Elvira et al. [40], which showed no positive results in associated psychopathology following treatment. Some of these secondary symptoms such as sleep could benefit directly or indirectly from the improvement of the main symptomatology of the disease, given that sleep problems are associated with impaired cognitive function [44] and attention, which is closely related to the processes of pain sensitisation and stimulatory inhibition [36].

## Conclusion

This review of the different types of EEG neurofeedback shows that the results of each study cannot be extrapolated due to their marked differences, i.e. they are not of general application to clinical or research contexts involving use of a different EEG-neurofeedback protocol to that of the study. Therefore, this systematic review helps to unify the results of different protocols to provide answers as to whether EEG neurofeedback is a current or potential treatment for psychological, physiological and health-related symptoms in fibromyalgia patients.

EEG neurofeedback is a relatively new field and there are few studies under chronic disease conditions specifying what works and in which conditions, i.e. defining the parameters of the training (duration and number of sessions, EEG-neurofeedback type, protocol type, monotherapy vs multitherapy). This review reveals that most of the studies were not controlled and did not use randomisation. Nonetheless, they can justifiably be described as ecologically valid because they did not use strict inclusion/exclusion criteria and selected fibromyalgia patients with a wide range of psychological, physiological and general health conditions. However, there is still a need for high-quality clinical trials under more randomised double-blind placebo-controlled designs such as those of Kravitz et al. [30] and Nelson et al. [33] to draw conclusions regarding the efficacy of clinical interventions for this type of patient. The latter authors do not recommend EEG neurofeedback (LENS) as a stand-alone therapy for fibromyalgia patients. To sum up, the lack of consistency of the EEG-neurofeedback protocols studied, the lack of homogeneity of the protocols and the different criteria for inclusion of the study participants demonstrate that the field has not advanced far enough to be able to draw conclusive results.

### Limitations

This review has certain limitations. The main limitation is that only quantitative articles and published doctoral theses have been considered, whilst Master’s theses [59, 60] and other types of scientific papers have not been taken into account.
